# The Treatment of Warty Growths of the Genitals

**Published:** 1897-02

**Authors:** 


### Abstracts and Selections.

### The Treatment of Warty Growths of the Genitals.

ABSTRACT BY THE AUTHOR.

Wiliam S. Gottheil, in a paper on Epithelioma of the Penis, read before the Society for Medical Progress, November 14th, 1896, concludes as follows (*International Journal of Surgery*, January, 1897):

1. Warty growths of the genitals, more especially in the male, are always to be suspected of malignancy, no matter how innocent they seem.

2. They should either be left entirely alone, or be thoroughly removed by knife or cautery.

3. Imperfect attempts at destruction, as with nitrate of silver, carbolic acid, etc., are especially to be avoided; there being many cases recorded in which they have apparently stimulated a benign growth into malignant action.

### CONTAGIOUS IMPETIGO.

(*Pediatrics*, October, 1896.) This is a self-limited contagious disease of children appearing in localised epidemics, and first described by Tilbury Fox in 1864. Accompanied by a moderate fever and some gastric disturbance, there appear on the face and hands groups of flat vesicles filled with transparent or cloudy serum. These dry up into characteristic golden-yellow crusts, which fall off in two or three weeks, leaving circular, reddened, non-ulcerated areas behind. Successive crops of vesicles may prolong the disease for two months or more. It is undoubtedly parasitic; but, though Kaposi claims to have found it, the etiological factor is still unknown. The treatment consists in removal of the crusts with olive oil compresses, cleansing the skin with hot water and soap, boric acid solution, etc., followed by the use of Lassar's paste:

℞ Acid. salicylic............................30 grains
Petrolati ..................................1 ounce
Zinci oxidi.................................
Amyli...............................a. a. ½ ounce

### SYPHYLODERMATA.

Abstract of Clinical Lecture delivered at the New York School of Clinical Medicine, November 25th, 1896.

A careful consideration and trial of the various methods of treating the syphilodermata has led me to the following conclusion:

1. In the primary stage, when only the chancre is present, no general treatment; calomel locally.

2. As soon as the secondary period sets in, as shown by the general adenopathy, angina, cephalalgia, and eruption, the internal treatment for mild cases should be ¼ to ¾ of a grain of the proto-iodide of mercury t. d., continued for three months, or until the symptoms disappear. In severe cases, with pustular eruptions, severe anginas, persistent headaches, etc., a course of six to ten intra-muscular injections of 10 per cent. calomel-albolene suspension, five to ten minims at intervals of five to fifteen days, should be employed.

3. After completion of the course and cessation of the symptoms, employ tonics, etc., without specific treatment, for three months.

4. Thereupon a second calomel course as above, plus a small dose (15 grains) of iodide of potassium in milk after meals. This to be given whether later secondary symptoms of the skin and mucosæ appear or not.

5. Second intermission of treatment, lasting three to six months, according to the presence or absence of symptoms.

6. In the second year, if tertiary lesions marked by deeper and more localised ulceration are present, give the iodide of potassium, increasing doses (60 to 600 grains daily) as may be necessary. Combine with it occasional courses of calomel injections. If no lesions appear, give a mild course of both.

The best local treatment of the syphilodermata is with the mercurial plaster-mull.

---

**Under** the head of "Browning and the Larger Public," the *Review of Reviews* for February publishes two articles of more than ordinary interest. Dean Farrar treats of "The Significance of Browning's Message," dwelling particularly on the poet's optimism, while the warden of "Browning Hall," in South London, Mr. F. Herbert Stead, writes on "Browning as the Poet of the Plain People." Mr. Stead declares that Browning has too long been regarded as the exclusive property of the learned. Too much stress has been laid on the obscurities of his style. The "Browning cult" has tended to discourage the popularizing of his poetry.

